# Presumed X‐Linked Retinoschisis in a 3‐Month‐Old Baby Girl: A Case Report

**DOI:** 10.1155/crop/2850410

**Published:** 2025-12-05

**Authors:** Hanieh Fakhredin, Esmaeil Asadi Khameneh, Shahin Faghihi, Mohammadreza Mehrabi Bahar, Fatemeh Bazvand

**Affiliations:** ^1^ Vitreoretinal Department of Ophthalmology, Farabi Eye Hospital, Tehran University of Medical Sciences, Tehran, Iran, tums.ac.ir

**Keywords:** bullous peripheral schisis, juvenile retinoschisis, RS1, X-linked retinoschisis, XLRS

## Abstract

**Purpose:**

The purpose of the study is to report a 3‐month‐old Iranian baby girl presenting with bilateral macular and peripheral retinoschisis with a probable diagnosis of X‐linked retinoschisis (XLRS).

**Methods:**

The baby underwent an ophthalmic examination under general anesthesia revealing bilateral vitreous hemorrhage, spoke‐wheel pattern radiating from the fovea, and severe bullous peripheral retinal elevation. We performed indirect scatter laser photocoagulation with a few spots, which showed a positive response confirming the diagnosis of retinoschisis over rhegmatogenous retinal detachment. Spectral‐domain optical coherence tomography (SD‐OCT) and intraoperative fluorescein angiography (FA) were also performed to enhance the diagnostic evaluation.

**Results:**

Based on comprehensive clinical examinations and imaging findings, XLRS was determined to be the most likely diagnosis.

**Conclusion:**

This case highlights the importance of including retinoschisis, with possible X‐linked inheritance, in the differential diagnosis of bilateral schisis and vitreous hemorrhage in female infants. Although the XLRS phenotype is rare in the female population, it appears to be more severe and manifests at an earlier age. Genetic confirmation is needed to determine the exact etiology.

## 1. Introduction

X‐linked retinoschisis (XLRS) is one of the most common forms of retinal degeneration in young males worldwide, with an estimated prevalence of 1 in 5000 to 1 in 20,000 [1]. The main diagnostic feature is the presence of a spoke‐like foveal schisis pattern, characterized by central radial streaks. Peripheral retinoschisis especially in the infratemporal region of the retina is detected in less than 50% of patients. The most commonly observed abnormality in full‐field electroretinography (ERG) is a reduced ratio of a‐to‐b amplitude or even electronegative ERG [1, 2]. The natural progression of the disease varies widely, even among family members, with vision ranging from near normal to complete blindness [1, 3]. Although the visual acuity of patients often remains stable over the years, complications such as vitreous hemorrhage, retinal detachment, and neovascular glaucoma are the main culprits of significant visual impairment [3].

Over 200 mutations of the Retinoschisis 1 (RS1) gene on chromosome Xp22 have been identified as causing XLRS, and as of now, no specific genotype–phenotype correlation has been established [4, 5]. The RS1 gene, responsible for encoding retinoschisin, is expressed by photoreceptors and bipolar cells and is believed to play a role in maintaining retinal structural integrity through intercellular adhesion and signal transduction between the cells [6]. Female carriers are typically asymptomatic and may only exhibit minor alterations on multifocal ERG [1, 3]. However, there have been reports of carriers displaying the typical XLRS phenotype [7]. Although XLRS is commonly diagnosed during school age, a few cases have been described in the first year of life [8, 9].

In this report, we present a case of probable XLRS in a patient with two distinctive characteristics—age and gender—that set it apart from previous case reports.

## 2. Case Presentation

A 3‐month‐old Iranian baby girl underwent a routine ophthalmologic examination at another center due to adoption. During the initial examination, bilateral vitreous hemorrhage was noted. After the vitreous hemorrhage resolved over subsequent follow‐ups, the infant was referred to our center with a diagnosis of bilateral retinal detachment. No information was available regarding the baby′s gestational age, birth weight, parental consanguinity, or family history. The caregivers did not report any history of trauma.

**Figure 1:**
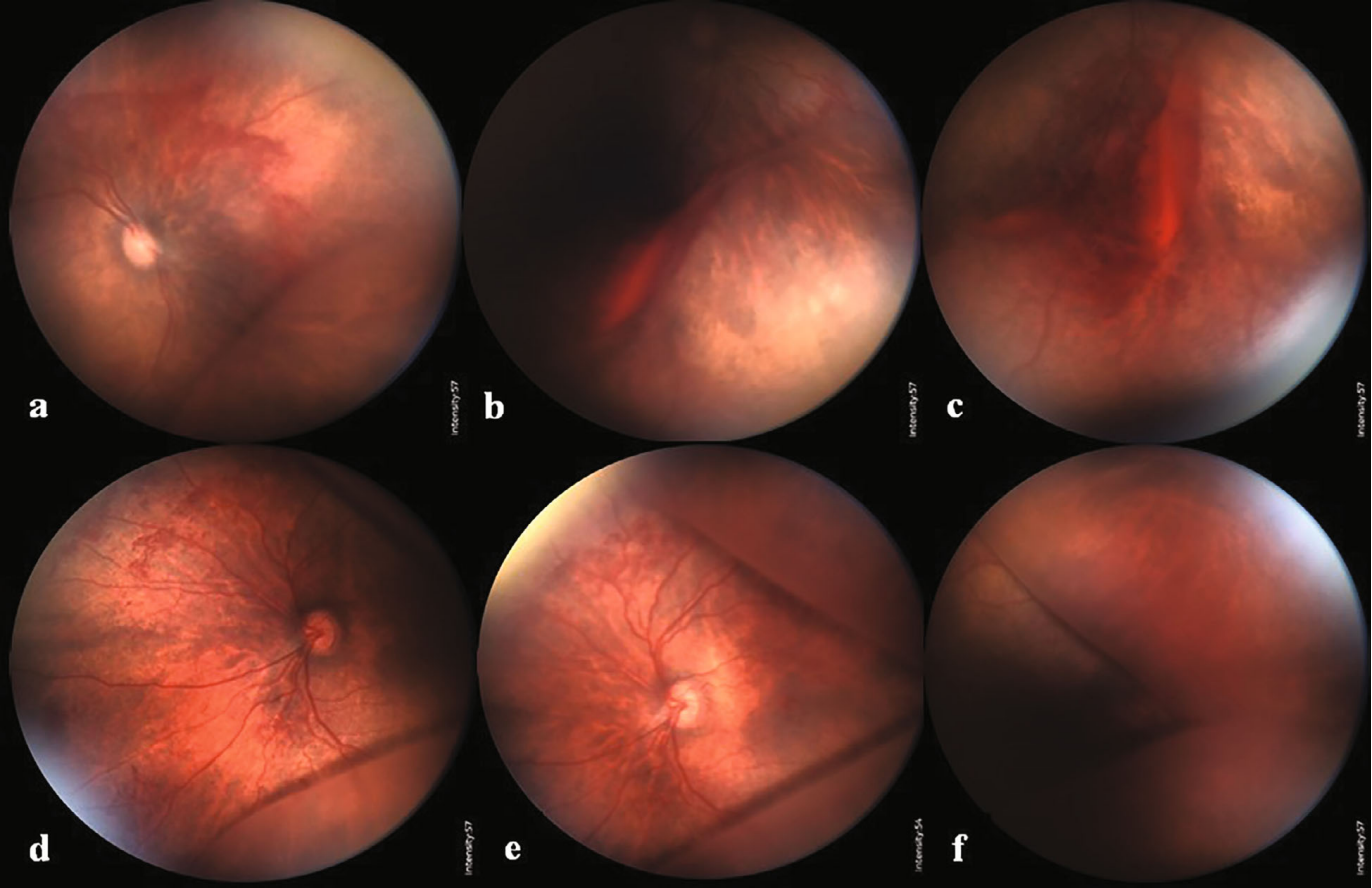
Fundus photographs taken by RetCam. (a–c) Right eye. (a) Intraretinal hemorrhage and peripheral retinoschisis in the inferonasal retina extending to the posterior pole. (b, c) Retinoschisis, preretinal hemorrhage, and organized vitreous hemorrhage in the periphery of the retina. (d–f) Left eye. (d, e) Intraretinal hemorrhage and bullous peripheral retinoschisis, which is extended to the posterior pole adjacent to the superior and inferior vascular arcades. (f) The bullous and dome‐shaped retinoschisis in the peripheral retina.

Upon ophthalmic examination under general anesthesia, the baby′s anterior segments were unremarkable. Fundus examination revealed normal optic discs and reduced foveal reflexes bilaterally. A spoke‐wheel pattern radiating from the fovea was observed, complicated by scattered predominantly preretinal hemorrhages (as re‐evaluated on fundus photographs) bilaterally. An increased distance between superior and inferior arcades and straightening of the vessels were also noted. Peripheral fundus examination in both eyes revealed severe bullous dome‐shaped retinal elevation with a smooth surface, which was more pronounced in the left eye, extending to the posterior pole adjacent to the vascular arcades. Preretinal hemorrhage and organized vitreous hemorrhage were also detected in the periphery of the right retina (see Figure 1).

**Figure 2:**
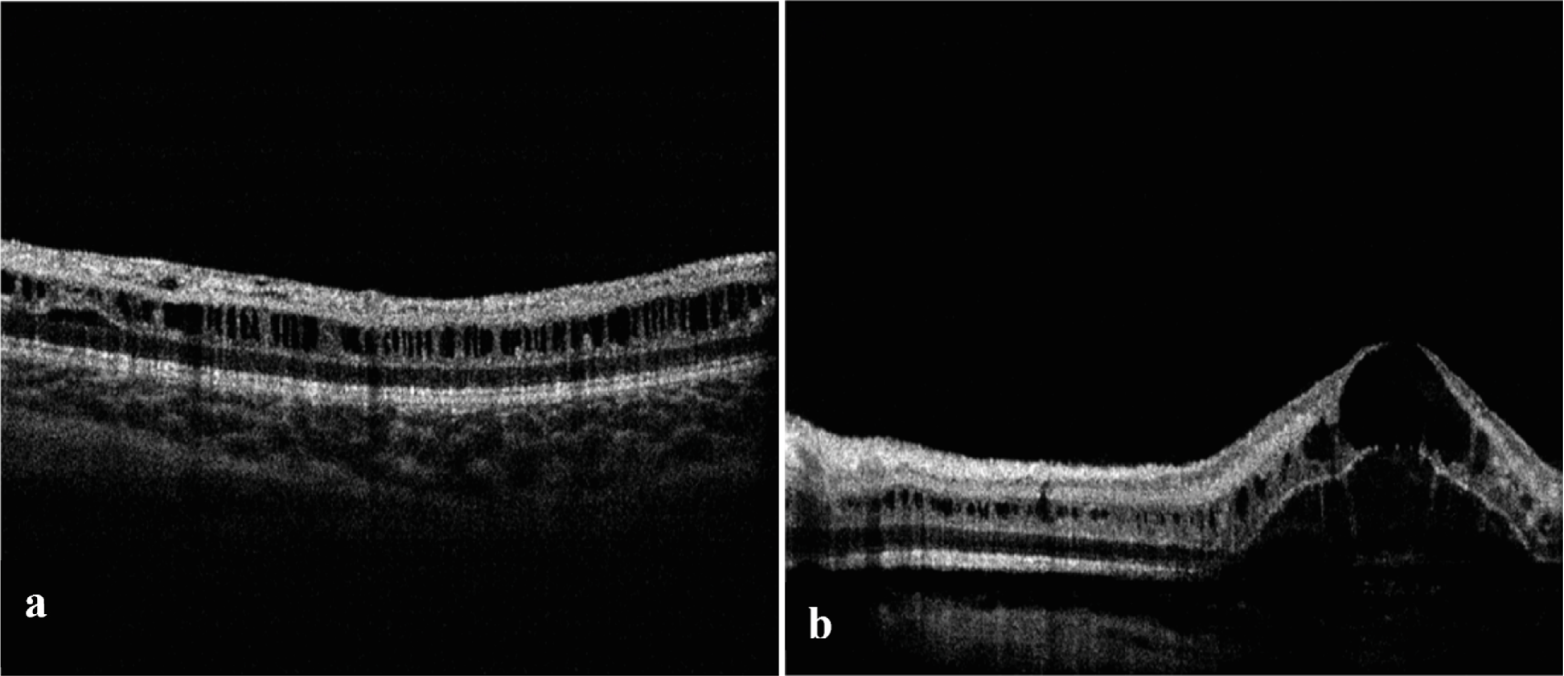
Spectral‐domain optical coherence tomography scans of the macular area. (a) Intraretinal cystic changes in the ganglion cell layer, inner nuclear layer, and outer nuclear layer. Macular schisis is evident in the inner nuclear layer in the right eye. (b) Severe foveal schisis in the inner and outer nuclear layers, which prevents viewing the details of the left fovea. The schisis in the inner nuclear layer extended from the fovea to the nasal macular area.

Given the clinical presentation, a retinoschisis was considered the most probable diagnosis. Indirect scatter laser photocoagulation with a few spots was performed, showing a positive response, which confirmed the diagnosis of retinoschisis over rhegmatogenous retinal detachment. Furthermore, the absence of a full‐thickness retinal break supports the exclusion of rhegmatogenous retinal detachment. Subsequently, spectral‐domain optical coherence tomography (SD‐OCT) using handheld portable SD‐OCT (Optovue iVue SD‐OCT Wellness report, Optovue Corporation, Fremont, California) was conducted, revealing macular schisis in the inner nuclear layer (INL) with intraretinal cystic changes located in various layers, including the ganglion cell layer, INL, and outer nuclear layer (ONL). These cystic changes extended beyond the foveal region, with more significant involvement observed in the left eye (see Figure 2).

**Figure 3:**
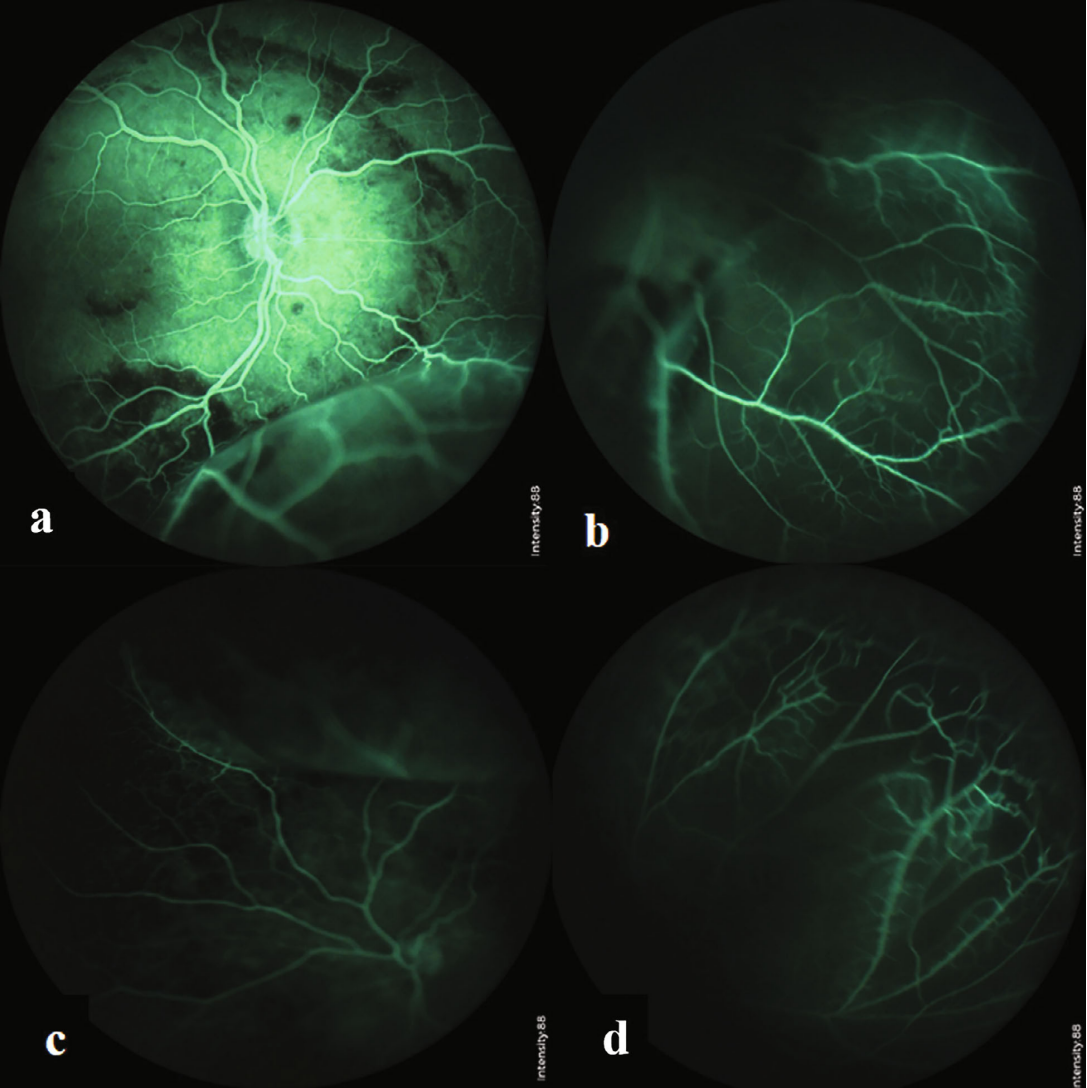
Fluorescein angiography images. (a, b) Right eye, hypofluorescent areas corresponding to the retinal hemorrhage are visible in the posterior pole. There is no macular leakage in the areas with macular schisis. (a) Areas with retinal hemorrhage are visible as hypofluorescent areas due to blockage. (c, d) Left eye, demonstrate blockage of choroidal hyperfluorescence due to bullous peripheral retinoschisis and certain nonperfusion areas as depicted in (d).

Intraoperative fluorescein angiography (FA) (using Phoenix Clinical ICON Paediatric Retinal Camera, Phoenix Technology Group Company, California, United States) revealed hypofluorescent areas corresponding to areas with retinal hemorrhage due to blockage of fluorescein. There was no vascular leakage in the macula of both eyes despite extensive macular schisis on the SD‐OCT images. In the peripheral retina, particularly in the areas with bullous retinoschisis, the FA demonstrated blockage of choroidal hyperfluorescence and better visibility of retinal vessels′ hyperfluorescence. The retina was completely vascularized without evidence of peripheral neovascularization (see Figure 3).

**Figure 4:**
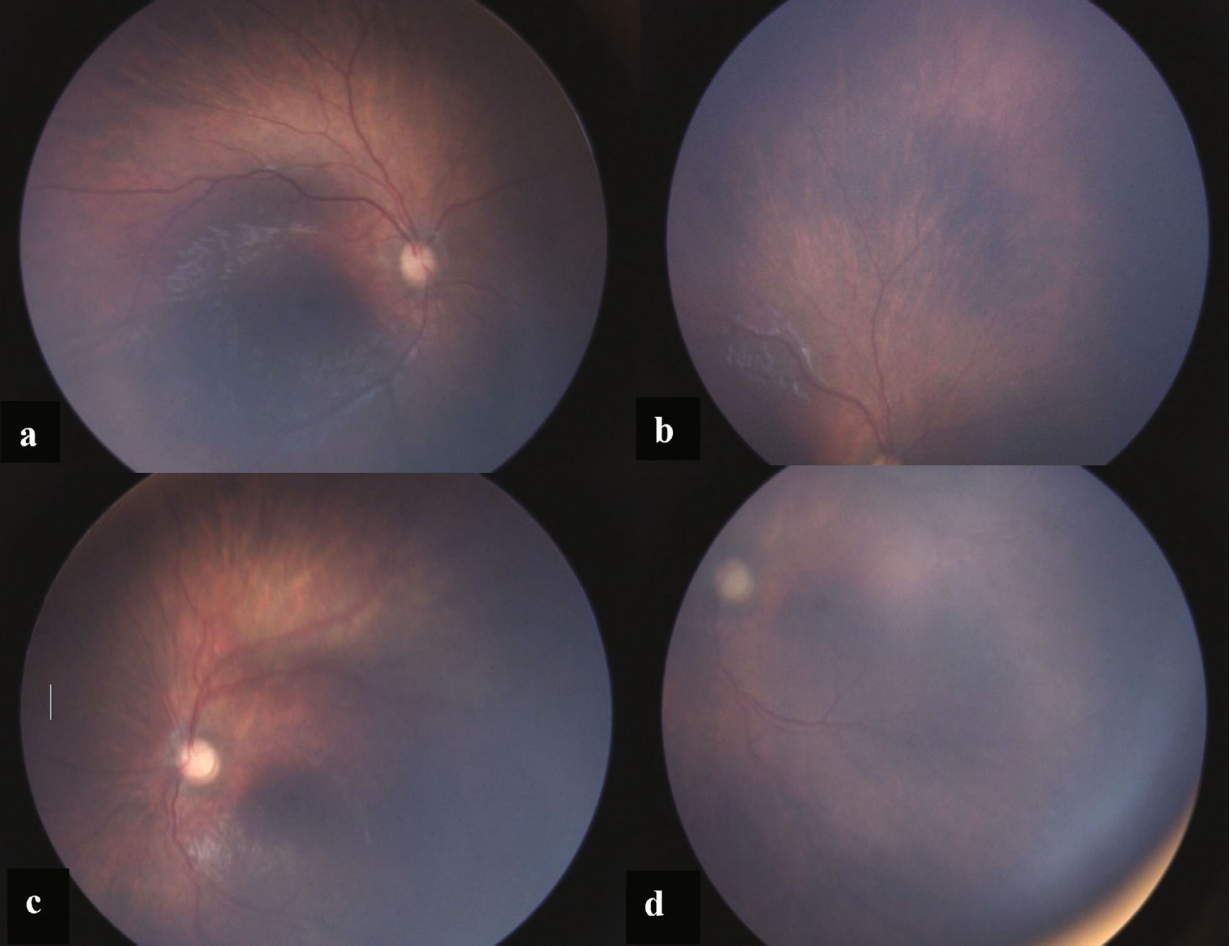
Fundus photos with RetCam after 1 year of follow‐up without any treatment. (a, b) Right eye. (c, d) Left eye. The photos show complete resolution of the intraretinal hemorrhage in posterior poles (a, c) and schisis in the periphery of the right retina (b). The height of the schisis in the inferior quadrants of the left retina has decreased significantly (d).

Based on the comprehensive clinical examinations and imaging findings, XLRS appeared to be the most likely diagnosis. However, it is important to note that the definitive confirmation of this diagnosis was pending molecular genetic analysis, which had not been conducted for the patient. The infant was placed under close follow‐up, and after 1 year without any specific treatment, the intraretinal hemorrhage resolved, and the extent of schisis was significantly reduced which supported our diagnosis in accordance with the possibility of self‐improvement observed in prior studies of XLRS [8, 9] (see Figure 4).

## 3. Discussion

The presence of vitreous hemorrhage and retinoschisis on fundus examination and multimodal imaging prompted consideration of several differential diagnoses. The first consideration was retinopathy of prematurity (ROP). However, due to insufficient data regarding the patient′s birth weight and gestational age, we were unable to definitively establish this diagnosis. Additionally, it is noteworthy that the pattern of tractional retinal detachment (TRD) is typically more pronounced in ROP than in retinoschisis. Furthermore, the normal pattern of retinal vascularity development observed in FA images did not align with a diagnosis of ROP [10].

Terson syndrome, while uncommon in pediatric populations, was considered a possible differential diagnosis. Potential causes include traumatic head injury, birth trauma, nontraumatic intracranial bleeding linked to leukemia, venous sinus thrombosis, and venous malformations. The diagnostic clue lies in the occurrence of subarachnoid or subdural hemorrhages alongside intraocular hemorrhage, particularly vitreous hemorrhage [11].

Abusive head trauma is an alternative diagnosis that could present with these clinical manifestations. Moreover, traumatic retinoschisis has been observed in around 8% of cases [12]. However, after a thorough evaluation by a pediatrician, which revealed no indications of head or body trauma or intracranial bleeding, these two diagnoses were ultimately excluded. Therefore, the diagnosis of XLRS became more prominent for us.

As previously mentioned, XLRS is a retinal degeneration inherited through an X‐linked recessive pattern, exhibiting complete penetrance and phenotypic variability [1]. Typically, this condition predominantly affects the male population; however, in rare instances, it can occur in females. The most plausible hypothesis to explain this occurrence, as outlined in several case reports, involves consanguineous marriage between an affected father and a carrier mother, which can lead to the birth of a clinically affected homozygous baby girl [13, 14]. However, since we lack information about the pedigree of this adopted baby, the applicability of this hypothesis is uncertain in our case.

The second hypothesis is the occurrence of the disease in female carriers due to the inactivation of one X chromosome, also known as “lyonization phenomenon.” As a result, even though carrier females are typically asymptomatic, they can exhibit a variety of phenotypes, sometimes more pronounced than those seen in hemizygous males [15].

The third hypothesis involves the birth of a girl with Turner syndrome, a condition characterized by the complete absence or dysfunction of an X chromosome (45XO). In this scenario, if the girl carries an X chromosome containing the RS1 mutation, she may develop the disease [16].

The fourth hypothesis suggests an autosomal inheritance model of the disease. As indicated by the study conducted by Vincent et al., biallelic mutations in CRB1 can lead to familial foveal retinoschisis [17].

In the present case, several distinctive clinical and imaging features support a presumed diagnosis of XLRS over other possible etiologies. The spoke‐wheel configuration of the macula, bilateral bullous peripheral schisis without any full‐thickness retinal breaks, and absence of vascular leakage on FA are all hallmark characteristics repeatedly reported in XLRS rather than in other forms of retinoschisis. In contrast, CRB1‐associated (autosomal recessive) retinoschisis usually presents with foveal‐limited schisis, sometimes accompanied by pigmentary changes or Coats‐like vasculopathy, none of which were observed in this patient [18]. Moreover, chromosomal abnormalities such as Turner syndrome may rarely mimic this phenotype, yet our patient demonstrated no systemic or dysmorphic features suggestive of such a syndrome.

The spontaneous partial resolution of schisis and intraretinal hemorrhage during follow‐up is also more consistent with the natural course of XLRS than with these alternative diagnoses.

Therefore, although molecular confirmation was not available, the constellation of clinical and imaging findings provides strong presumptive evidence for XLRS.

In accordance with the study conducted by Murro et al., it has been demonstrated that the severity of the initial manifestation of XLRS plays a significant role in the timing of diagnosis. For instance, when the initial symptoms are more severe, such as vitreous hemorrhage or extensive bullous peripheral schisis involving the posterior pole, the diagnosis tends to occur sooner. Conversely, milder symptoms, such as intraretinal hemorrhage or small peripheral schisis, often lead to a later diagnosis [19]. It is worth noting that the disease′s tendency to be diagnosed during school age may be attributed to the milder phenotype and the complaints of reduced vision expressed by children at this age.

For reasons not yet fully understood, XLRS tends to manifest more severely in affected females, leading to earlier detection of the disease. This aligns with the case of our 3‐month baby girl, whose initial manifestation was vitreous hemorrhage. This may be due to the presence of two different types of severe mutations in X chromosomes or the presence of two defective alleles [20].

While the clinical and imaging findings are highly suggestive of XLRS, the absence of genetic testing prevents a definitive diagnosis and investigation of inheritance patterns. Rare reports have described atypical or pseudodominant patterns of XLRS, usually resulting from consanguinity or lyonization rather than true dominant inheritance [21]. Other possibilities, such as autosomal retinoschisis (e.g., CRB1‐associated), should still be considered in the differential.

This case highlights the importance of recognizing XLRS‐like phenotypes even in atypical demographics and underscores the need for future genetic studies. Documenting such rare presentations may help guide genetic counseling and promote inclusion of similar patients in future gene therapy trials targeting the RS1 pathway [20].

It is crucial to acknowledge the limitations of our study, particularly the lack of genetic testing to confirm the diagnosis definitively. Genetic analysis of the RS1 gene was not feasible due to the prohibitive costs of testing that are not covered by national insurance, coupled with the absence of consent from the adoptive guardians. Furthermore, given the baby′s age of 3 months, we refrained from conducting an ERG due to its limited reliability at this developmental stage. Despite these limitations, the clinical diagnosis remains strongly supported by characteristic multimodal imaging findings, a positive response on indirect scatter laser photocoagulation, the absence of alternative compatible diagnoses, and spontaneous partial resolution on follow‐up. Thus, this case retains significant value as one of the earliest and rarest documented presentations of presumed XLRS in a female infant. Future research should focus on elucidating the underlying genetic mechanisms contributing to XLRS in atypical cases, paving the way for improved diagnostic and therapeutic strategies tailored to individual patients.

## 4. Conclusion

This case highlights the importance of including retinoschisis, with possible X‐linked inheritance, in the differential diagnosis of bilateral schisis and vitreous hemorrhage in female infants. Although the XLRS phenotype is rare in the female population, it appears to be more severe and manifests at an earlier age. Genetic confirmation is needed to determine the exact etiology.

**Nomenclature**
ERG: electroretinography
FA: fluorescein angiography
INL: inner nuclear layer
ONL: outer nuclear layer
RS1: Retinoschisis 1
SD‐OCT: spectral‐domain optical coherence tomography
XLRS: X‐linked retinoschisis

## Ethics Statement

This manuscript adheres to the tenets of the Declaration of Helsinki.

## Consent

Written informed consent was obtained from the patients′ legal guardians for publication of this case report and any accompanying images. A copy of the written consent is available for review by the Editor‐in‐Chief of this journal.

## Disclosure

All authors read and approved the final manuscript.

## Conflicts of Interest

The authors declare no conflicts of interest.

## Author Contributions

All authors contributed to the study conception and design. All authors commented on previous versions of the manuscript.

## Funding

No funding was received for this manuscript.

## Data Availability

The patient data utilized in this study are not publicly available due to privacy concerns. However, they can be obtained from the corresponding author upon reasonable request.
